# Sodium Salicylate in Scarlatina

**Published:** 1916-05

**Authors:** 


**Sodium Salicylate in Scarlatina.** Felix Raymond, Soc. Med. des Hop., Oct. 22, 1915, advocates this as a routine treatment, the initial dose being 2 grams, then 6 grams a day in ½ gram doses, with due regard to individual susceptibility. Immediate amelioration occurs and complications are avoided.

